# The AIDS and Cancer Specimen Resource: Role in HIV/AIDS scientific discovery

**DOI:** 10.1186/1750-9378-2-7

**Published:** 2007-03-02

**Authors:** Leona W Ayers, Sylvia Silver, Michael S McGrath, Jan M Orenstein

**Affiliations:** 1 Department of Pathology, The Ohio State University, Columbus, Ohio, USA; 2 Department of Pathology, The George Washington University, Washington DC, USA; 3 Department of Pathology, University of California, San Francisco, California, USA

## Abstract

The AIDS Cancer and Specimen Resource (ACSR) supports scientific discovery in the area of HIV/AIDS-associated malignancies. The ACSR was established as a cooperative agreement between the NCI (Office of the Director, Division of Cancer Treatment and Diagnosis) and regional consortia, University of California, San Francisco (West Coast), George Washington University (East Coast) and Ohio State University (Mid-Region) to collect, preserve and disperse HIV-related tissues and biologic fluids and controls along with clinical data to qualified investigators. The available biological samples with clinical data and the application process are described on the ACSR web site.

The ACSR tissue bank has more than 100,000 human HIV positive specimens that represent different processing (43), specimen (15), and anatomical site (50) types. The ACSR provides special biospecimen collections and prepares speciality items, e.g., tissue microarrays (TMA), DNA libraries. Requests have been greatest for Kaposi's sarcoma (32%) and non-Hodgkin's lymphoma (26%). Dispersed requests include 83% tissue (frozen and paraffin embedded), 18% plasma/serum and 9% other. ACSR also provides tissue microarrays of, e.g., Kaposi's sarcoma and non-Hodgkin's lymphoma, for biomarker assays and has developed collaborations with other groups that provide access to additional AIDS-related malignancy specimens. ACSR members and associates have completed 63 podium and poster presentations. Investigators have submitted 125 letters of intent requests. Discoveries using ACSR have been reported in 61 scientific publications in notable journals with an average impact factor of 7.

The ACSR promotes the scientific exploration of the relationship between HIV/AIDS and malignancy by participation at national and international scientific meetings, contact with investigators who have productive research in this area and identifying, collecting, preserving, enhancing, and dispersing HIV/AIDS-related malignancy specimens to funded, approved researchers at no fee. Scientific discovery has been advanced by this unique biorepository. Investigators are encouraged to browse the ACSR Internet site for materials to enhance their own scientific initiatives.

## Review

### Background

Human Immunodeficiency Virus (HIV) infection continues its global spread with almost 40 million HIV-infected individuals worldwide. More than half are women [1]. While Acquired Immunodeficiency Syndrome (AIDS) is most associated with opportunistic infections, adults and children infected with HIV have well-recognized excess risks for the development of malignancies [2]. Many of these cancers are associated with known oncogenic viruses such as: Epstein Barr virus (EBV) with non-Hodgkin's lymphoma, human herpes virus 8 (HHV8) with Kaposi's sarcoma (KS), Hepatitis B (HBV) and Hepatitis C (HCV) viruses with hepatocellular carcinoma and human papilloma virus (HPV) with squamous cell carcinomas of cervix and anal canal. Other cancers, such as lung, are being studied for their relationship to HIV infection. In the developed world, cigarette smoking is common and HIV infected smokers have an increased incidence of smoking-related cancers of the lip, mouth, and pharynx [3]. With the advent of highly active antiretroviral therapy (HAART) in 1996, there was a dramatic reduction in the incidence of non-Hodgkin's lymphomas (NHL) and especially KS. However, apparently little reduction is suggested for the other excess risk cancers including Hodgkin's lymphoma, squamous cell carcinoma of anus, multicentric Castleman's disease in adults and leiomyosarcoma in children. Despite HAART and public education the total number of individuals living with HIV in the United States continues unabated with more than a 50% increase since 1996. HIV associated cancers in the HAART era are still among the top causes of death in HIV/AIDS [4]. Furthermore it is of concern that extended exposure of such a large population of HIV infected individuals of all ages to nucleoside analogs such as azidothymidine (AZT) and dideoxycytidine (DDC), as well as, other potent AIDS drugs may induce DNA damage leading to other significant associations with cancer in the future.

The need to obtain and provide tumor tissue from patients with AIDS was originally identified at a conference on AIDS Lymphoma in May, 1992. The request for a resource for such specimens was made both by investigators participating in clinical trials research and laboratory investigators. Thus the National Cancer Institute (NCI) established the unique AIDS Malignancy Bank (AMB) in 1994 as a cooperative agreement to collect HIV positive and control biological specimens, with associated clinical data, to support translational research into HIV/AIDS-related malignancies [5]. This AMB program continues as the AIDS and Cancer Specimen Resource (ACSR) [6], supporting broadened research interests in the area of HIV/AIDS and oncogenic viruses [7] through collaborative specimen assemblage. HAART associated reductions in certain HIV/AIDS-related malignancies make these specimens more difficult to acquire (e.g., KS and NHL), therefore the ACSR is a critical conduit for the collection, preservation, banking and disbursement of scarce HIV/AIDS-related malignant tissues and biological fluids to qualified investigators.

In addition to serving as a resource for specimens acquired from the consortia sites, the ACSR is a national repository of specimens that reflects the temporal changes, and thus the history, associated with HIV disease. During the course of the last decade, the ACSR has established itself as a singular specialized resource for collection, storage, and provision of specimens with associated clinical, diagnostic, and epidemiologic data collected from HIV-infected individuals participating in clinical trials, observational cohort studies, and other research studies conducted by AIDS investigators both domestically and internationally. During the life of the ACSR, the biorepository cost of specimens has decreased significantly making the ACSR a good value to support research by RO1/R21 funded investigators engaged in HIV/AIDS malignancy studies.

### Specimens within the ACSR

The ACSR bank has more than 100,000 specimens from HIV infected and control patients available from different processing (43), specimen (15), and anatomical site (50) types. Specimens are representative of the HIV/AIDS evolving epidemic and are from a wide variety of patients, including those with early, as compared to late-stage disease, multi-site autopsy specimens with involved and uninvolved tissue, and specimens obtained from patients with defined anti-retroviral or chemotherapy histories. In addition, the inventory contains donations from the AIDS epidemics in developing countries outside the United States.

### Special emphasis specimen and data acquisitions

• *Detailed viral typing studies*. The ACSR has expanded tissue collection at international sites including Africa, Brazil, and Thailand to obtain specimens from patients who develop malignancies associated with infection from different subtypes of HIV, HHV-8, EBV, and HPV. These types of specimens are critical for determining the role of virus associated carcinogenesis in the natural epidemic setting, as opposed to clinical settings where there is anti-viral therapy intervention.

• *Neoplastic and non-neoplastic specimens from women*. Gynaecologic tissues, breast, and obstetrical specimens from HIV-infected women come from collaborative efforts with ongoing clinical studies of HIV-disease in women, i.e. Women's Interagency HIV Study (WIHS). Expansion of this collection to include specimens for recently discovered agents and unusual malignancies associated with HIV-disease is ongoing. The coordinated study between WIHS and the Rwanda Women's HIV Study, provides fresh gynaecological specimens (LEEPs, uterine resections, and biopsies) and biological fluids from infected women including normal tissue through cervical cancer.

• *Specimens from AIDS-related malignancies clinical treatment trials*. AIDS-related malignancy clinical trial groups (ACTG) obtain specimens that are collected and stored longitudinally. Samples retained from the early clinical trials are now available through the ACSR to approved investigators.

• *Specialty sub-bank samples*. Disease specific (lymphoma, KS) tissue micro-arrays (TMAs) for rapid assessment of tissue molecular targets or reagents (antibody, *in situ *probes, etc) are available. Specimens preserved specifically for transmission electron microscopy are available and provide optimal samples to carry out ultrastructural studies of malignant, infectious, and reactive tissue processes. Specimens preserved in fixatives recently shown to enhance immunohistochemical and *in situ *hybridization results are currently available or could be developed by request of approved investigators.

• *ACSR data interface*. The ACSR has developed a national computerized database of specimens with patient information and clinical history. Over time, this system will interface with electronic databases of other cooperative banking groups, as well as with clinical trial groups. A version of the ACSR database, stripped of patient identifiers, is made available online; researchers can search for useful specimens using various patient, specimen and clinical criteria.

### Investigator use of ACSR samples and clinical data

Investigators made 148 specimen availability inquires to the ACSR, 88 resulted in *full *applications and 37 in *short *applications. Of the full applications, 73 were approved to receive multiple samples, 64 received samples, 8 are still waiting funding or identification of appropriate specimens, and 14 were not approved for a variety of reasons including scientific merit, sample request size, or lack of available specimens. The latter investigators were referred to other bio-repositories or resources, when appropriate. Of the full approved applications, 83% were for tissue, 18% for serum/plasma and 9% for cell lines, bone marrow, or urine. Some applications were approved for mixed sample types. Of the 37 short form applications received, 36 were approved. The 23 remaining inquires included 19 for which no final application for samples was received and researcher inquires that were referred to other biological sample resources. Requests for Kaposi's sarcoma specimens accounted for 32% of the total served requests, lymphoma for 26%, and the remainder were for a variety of other disease types and controls.

Investigators have used the ACSR's HIV infected and non-infected control biological samples and clinical data to contribute significant discoveries. Included are: markers of selective B cell activation during lymphomagenesis [8], effect of HIV integration site on cancer development [9], role of macrophages [10], chemokines, cytokines and growth factors in cancer [11], KHV induced transcriptional reprogramming in KS cell types [12], correlation of interleukin, CD4+ lymphopenia, viral load and disease progression [13], persistent infections associated with cancer, especially EBV [14] and human papillomaviruses [15] and diagnostic assay development and validation [16].

Investigations into the pathobiology of HIV infection have used human tissue to provide translation from important discoveries made in cell culture or animal studies. HIV insertion sites within human tissues including somatic cells [17], macrophages and malignancies [18] have been defined using ACSR provided infected human tissue. The neuropathogenesis of AIDS dementia was explored [19] using HIV infected brain tissue and technical resources within the ACSR. Likewise human associated vasculopathy [20] and cardiomyopathy were elucidated by translation to HIV/AIDS human tissues relevant findings from a "murine AIDS" model [21].

**Figure 1:**
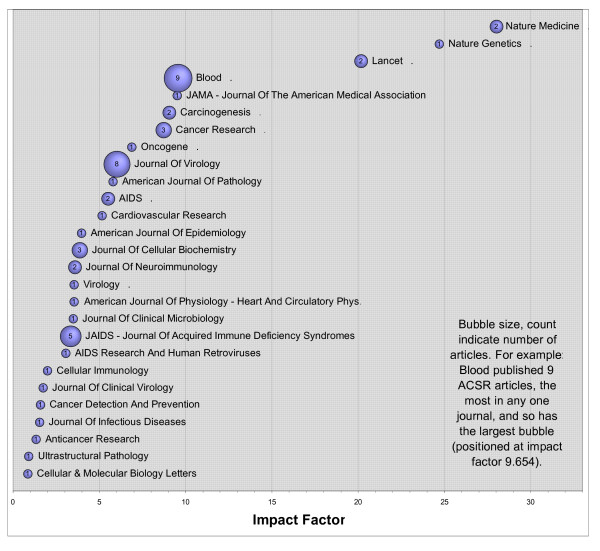
**Impact of ACSR Articles**. Journals that published ACSR related articles are shown with the bubble size representing the number of articles (count shown inside bubble) while the width represents the weighted average impact factor. For example, Nature Medicine, which published two ACSR articles, is shown at far right because it has the highest impact factor, 28.010.

The use of ACSR resources has resulted in sixty-one scientific reports added to the scientific literature and others are in preparation. Appendix I contains the list of these reports. Table 1 lists all publishing journals and Figure 1 depicts impact factors for them. Eighty-three podium and poster presentations have been made at local, national and international scientific meetings through 2006 by investigators using/promoting ACSR materials. Appendix II contains a list of these presentations.

**Table 1:** Journals publishing ACSR articles

*Journals*	Weighted*Impact Factor^#^	*Number of Articles*
Blood	9.654	9
Journal Of Virology	6.034	8
JAIDS – Journal Of Acquired Immune Deficiency Syndromes	3.345	5
Cancer Research	8.727	3
Journal Of Cellular Biochemistry	3.868	3
Nature Medicine	28.010	2
Lancet	20.158	2
Carcinogenesis	9.067	2
AIDS	5.517	2
Journal Of Neuroimmunology	3.598	2
Nature Genetics	24.695	1
JAMA – Journal Of The American Medical Association	9.522	1
Oncogene	6.872	1
American Journal Of Pathology	5.796	1
Cardiovascular Research	5.164	1
American Journal Of Epidemiology	3.978	1
Virology	3.540	1
American Journal Of Physiology – Heart And Circulatory Physiology	3.539	1
Journal Of Clinical Microbiology	3.503	1
AIDS Research And Human Retroviruses	3.069	1
Cellular Immunology	1.988	1
Journal Of Clinical Virology	1.744	1
Cancer Detection And Prevention	1.599	1
Journal Of Infectious Diseases	1.547	1
Anticancer Research	1.331	1
Ultrastructural Pathology	0.918	1
Cellular & Molecular Biology Letters	0.873	1
*Articles in journals with impact factors*	7.295	55
BMC Medical Informatics And Decision Making		2
Anatomical Record (Part B: New Anat.)		1
Annals Of Diagnostic Pathology		1
Current Opinion In Investigating Drugs		1
Humana Press		1
*Articles in journals without impact factors*		6
*Total articles*		61

* The impact factor of a journal varies by year. Each weighted impact factor is calculated by averaging the impact factor from the year of publication of each article.

^# ^As impact factors are calculated from citations in a three year window following publication date, the last published impact factor is used for some more recent articles.

### Developments within the ACSR

#### Evaluation of HIV DNA in ACSR specimens

The ACSR tested the suitability of: banked tissues to evaluate *in vivo *HIV sequences using an HIV bioinformatics facility. HIV sequence analysis was performed using the HIVbase^® ^software (GeneJohnson, Inc., St. Augustine, FL). HIV DNA was identifiable (at copy numbers greater than 1 per 10,000 cells) in:

a) >50% of blood derived T cell preparations

b) >33% of blood CD14+ monocytes

c) AIDS lymphomas

d) AIDS lymphoid tissue

e) kidney and seminal vesicle tissues, and

f) >90% of brain tissues from patients with HIV dementia.

Studies of HIV gene sequence selection/evolution in human HIV infected tissues from patients with AIDS malignancies as compared to other classes of HIV related disease can therefore be facilitated by ACSR resources [19]. The ACSR has recently begun the development of an AIDS lymphoma DNA library that can be made available to interested and qualified investigators.

#### Tissue micro-arrays from ACSR specimens for translational research

Tissue micro-arrays (TMA) [22] are useful for a wide range of research interests. One TMA tissue section can provide diagnostic tissue core sections (0.6 mm in diameter or greater) from literally hundreds of specimens, e.g., KS and NHL, from HIV-positive and negative individuals, for simultaneously testing. Immunohistochemical staining and *in situ *hybridization techniques are routinely employed on TMAs. In general, all ACSR TMAs also contain positive cores (e.g., cell culture lines) and negative control cores along with the disease specific cores allowing validation of novel tissue biomarkers. The ACSR provides non-Hodgkin's lymphoma and KS TMA slides to approved researchers interested in characterizing these tumors using applicable tissue probes. Other TMAs are planned, e.g., various subtypes of lymphoma characterized by immunohistochemistry and DNA.

**Figure 2:**
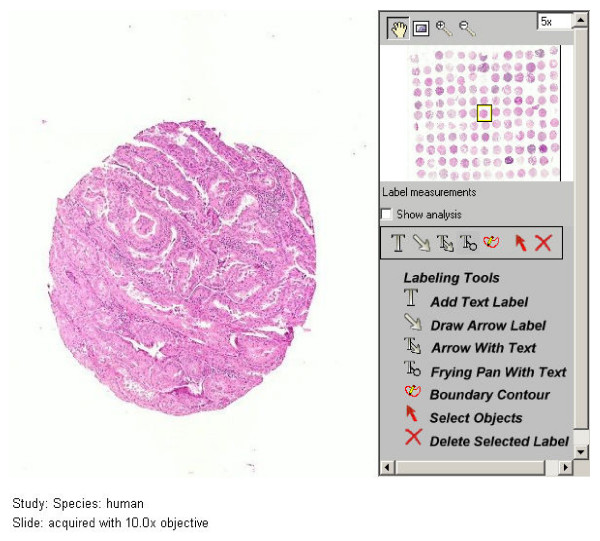
Sample TMA image from the virtual microscope section on the ACSR Mid-region Internet site for viewing TMS core tissues by interested investigators.

Preview and selection of TMAs by researchers are enhanced by the development and deployment of virtual whole slide images of digitized TMA stained sections and associated clinical data on an ACSR Internet site [23]. Online TMA and tissue core images (as in Figure 2), legends or data maps, and clinical data are linked for convenient browsing [24,25]. Data is available in an industry standard XML export format [26]. Printed data maps and images of TMA slides are also available. TMAs are offered as an enabling technology to expedite transitional research in HIV/AIDS and particularly in HIV-related malignancies, while conserving valuable specimens.

The quality of each TMA is directly related to the individual donor tissue core characteristics. Older archived paraffin embedded tissues may be antigen negative or weakly reactive and require optimal antigen retrieval methods and high antibody titres for positive results. Core donor tissues specifically prepared by optimal fixation and processing provide the most favourable TMA sections for studies with novel probes. Donor tissue characteristics are available with each TMA to provide guidance to the investigator. Cut sections from each TMA are stained by H & E to assure the presence of target tissues and by appropriate immunohistochemical stains (IHC) to assure tissue immunoreactivity for expected molecular targets prior to listing TMA availability on the Internet site.

### Structure

#### Organization of the ACSR

**Figure 3:**
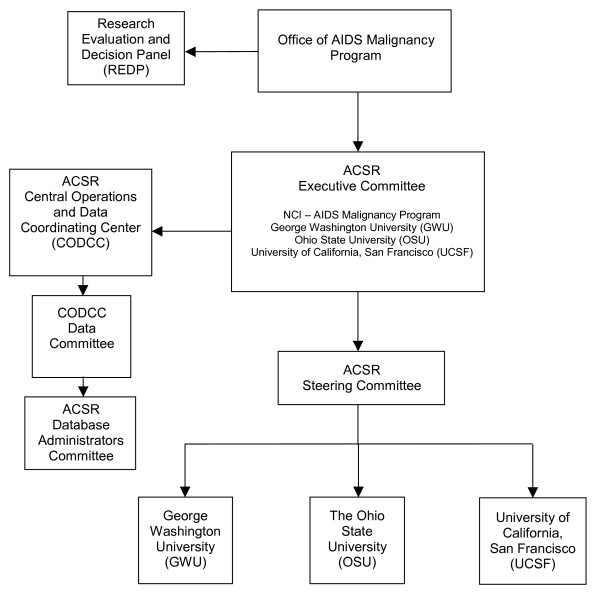
ACSR organization.

The national ACSR was formed as a cooperative agreement between the NCI, Office of the Director, Division of Cancer Treatment and Diagnosis (DCTD) and regional ACSR consortia: University of California, San Francisco for the West Coast, George Washington University for the East Coast and The Ohio State University for the Mid-Region. The ACSR steering committee, comprised of the principal investigators from each consortium, developed policies and procedures for standardized operation across the organization. An outside Research Evaluation and Decision Panel (REDP), comprised of experts in the field, evaluates requests for specimens as shown in Figure 3. See [6] for a detailed description of the program.

#### Privacy, Confidentiality, Authorization and Consent

Local Institutional Review Boards (IRBs) approve and monitor the activity of each ACSR site. The local ACSR maintains a research protocol to protect biologic sample donor privacy and to maintain confidentially of clinical data as required by federal standards including the requirements of HIPPA [27]. Local IRB approval is required for participation in the national ACSR program.

#### ACSR Internet site and national database

The ACSR established a database containing information on ACSR specimen types and associated clinical data obtained from patients with HIV/AIDS-related malignancies and control specimens. Each consortium sends electronic de-identified data quarterly to the central ACSR database. Records are maintained at each local site in accordance with the local IRB approved protocol. Some specimen collections have other databases that enable investigators to perform studies on specimens linked with a defined clinical or epidemiologic pedigree.

### Guidelines

The ACSR has quality practices for biorepository operation that are consistent with International Society for Biological and Environmental Repositories (ISBER) best practices [28], NCI Office of Biorepositories and Biospecimen Research (OBBR) guidelines [29] and AIDS Clinical Trials Group (ACTG) Biomedical Research Institute (BRI) guidelines [30].

### Marketing

The ACSR has strategies to market the availability of HIV/AIDS-associated malignancy specimens. Through word of mouth, directed mailings, newsletters, and presence (handouts and a specially designed ACSR booth) at national and international meetings, the ACSR reaches increasing numbers of potential researchers interested in utilization of the resource and have steadily increased the number of applications. The ACSR seeks feedback from its investigator community to identify future needs, gaps, and shortcomings.

### Specimen disbursement

#### Identifying researcher needs

Investigators search on-line (using the *Specimen Search *web page [6]) to identify specimen types that meet their research requirements. Once researchers find specimens of interest, they fill out a Letter of Intent (LOI) application for acquiring the specimens. The ACSR has two application types (short and long form) depending on the number of specimens needed. All of the requirements for researchers are available on the above Internet site. If the researcher is unsure of whether needed samples and data are available or has other questions regarding specimen and data availability, the ACSR or NCI program director can be contacted. Some of the general potential uses of ACSR specimens are outlined in Table 2.

**Table 2:** Potential uses of ACSR banked specimens

*Type*	*Use*	
Autopsies (multi-site) frozen & fixed	DNA and protein studies within individuals; involved vs. uninvolved tissues	
	
	Tissue array analyses	Comparison of antigen expression between many patient tumors
	
		Diseased tissue specific cytokine, virus, antigen expression

Frozen lymphoma, KS and tumor tissues	DNA, RNA, and protein array studies; viral discovery/strain variation studies	

Non Hodgkin's lymphoma (AIDS & non-AIDS) epidemiology study (serum & fixed tissue)	Serologic studies on cases vs. controls for cytokine, viral antigen, serum proteins. Coupled with epidemiologic data in collaboration with Dr. Elizabeth Holly test disease associations, risk factors, transmission risk factors, resistance factors, lymphoma tissue, DNA/protein correlation with serum factors/disease associations.	

AIDS Malignancy Consortium clinical trial specimens	Longitudinal, trial associated specimens for analyses of disease specific markers in collaboration with AMC	

Serum specimens from cross-sectional survey in Thailand	Studies on low KS prevalence untreated HIV & serum cohort Infected vs. uninfected age & sex matched specimen comparisons	
	
	Studies on HIV, non-US viral isolates. Repeat blood draws from HIV+ individuals available for rate of variation studies.	

Ano-genital specimens from men and women HIV+/HIV-	Study role of HIV strains in early stages of ano-genital carcinogenesis	

Plastic embedded tissue suitable for transmission electron microscopy	Evaluation of virus identity, morphogenesis, and cytopathology in various disease states	

#### Letters of Intent and tracking

Full and short form LOI applications are accepted from investigators throughout the year. Forms can be downloaded from the *Letter of Intent Application Form *web page [6]. The application process requires minimal paper work and is outlined in detail on the web site. When specimens are approved for delivery, they are packaged and shipped (on dry ice, when appropriate) according to International Air Transport Association (IATA) regulations. The ACSR works with other biological material resources and refers investigators to these resources if specimens are not available within the ACSR cooperative group. The confidential ACSR LOI tracking system allows inquiry tracking, providing details of requests and the status of shipments without disclosing research directions outside of the ACSR staff.

#### Sample Quality control

Investigators who receive ACSR specimens assist in the ACSR Quality Assessment Program by evaluating the quality of specimens they receive and returning a written evaluation document to the ACSR. For example, users have reported that blood stored by the ACSR yielded >90% high quality RNA and DNA and viability of PBMCs exceeding 50% in samples stored for up to five years. A return evaluation card is included with each sample shipment.

Random samplings of specimens are regularly tested for preservation of RNA and DNA and the viability of cells in frozen storage. Many of the banked specimens, however, are too small to allow extensive pre-testing that requires destructive sampling. The quality of stored biological samples can generally be anticipated based on specimen type, type of fixative or processing, length of storage, storage method and the type of testing anticipated by the investigator [31,32]. Investigators are encouraged to verify the use of specified biological sample types before undertaking their planned study.

#### Cost to investigators

Samples are provided to approved, funded investigators working in non-profit research settings at no fee. Investigators working in a commercial setting may also obtain samples if approved by the REDP but a fee per sample and for service may be applied.

### Specimen collection and processing

Specimens from a wide variety of HIV infected patients and selected controls are processed based on laboratory standard protocols described in the ACSR standard operating procedure manual, accessible through the *Process Specimens (Protocols) *web page [6]. This manual provides instructions for the collection, preparation and shipment of specimens among the ACSR sites and to approved investigators. Cooperative groups interfacing with the ACSR can use the ACSR manual as a resource for their activities.

### Specimens donated individually

Each ACSR site has developed a consortium of pathologists and clinicians at participating sites who collect and contribute specimens and clinical information to the ACSR. If samples are specifically collected for the ACSR, each donor signs an ACSR informed consent document and a HIPPA document approved by the local IRB. Samples from archived paraffin embedded tissues and associated clinical data are acquired according to local hospital rules, IRB approved protocols including federal HIPPA waiver of informed consent for use of personal medical information preparatory to research [27]. All samples and clinical data are coded when they are entered into the local ACSR site, de-identified before being placed on the web and all samples and clinical data released to investigators are anonymous.

### Samples donated by HIV/AIDS treatment and epidemiology groups

The ACSR is directly affiliated with the AIDS Malignancy Consortium (AMC) [33], a national group that conducts clinical trials in patients with AIDS-related malignancies. This affiliation allows the banking of well-characterized, longitudinal specimens from a variety of clinical trials for ultimate use by approved researchers. This association provides material for the search for prognostic markers and promising therapeutic regimens. Using the AMC relationship as a model, further group affiliations have been developed, such as relationships with the Women's Interagency HIV Study (WIHS), the AIDS Clinical Trials Group (ACTG), the San Francisco gay men's health study, the national ano-genital cancer study, San Francisco lymphoma study of 1600 NHL patients and 2500 random controls, natural history study of HHV8/KSHV in homosexual men in the San Francisco area, the Rwanda HIV women's study, and a variety of smaller studies. The WIHS is a unique study, which provides interval blood specimens and fresh colposcopy specimens from 2809 HIV-positive and 959 negative women who have cervical lesions. This study provides valuable pre and post diagnostic biologic specimens.

### Specimen collections donated or accessed through a referral process

Where large collections of specimens are in place within established programs, the ACSR can act as a broker between the ACSR research applicant and the "resource" tissue bank. Where there are biologic sample collections that have been placed at risk because of funding loss, tissue bank down sizing or other reasons, the ACSR can accept transfer of banked biological sample collections with their attendant databases, for inclusion in the overall ACSR program. Examples of the first type of relationship includes: interfaces with the National Neuro AIDS Bank (NNAB), the National Neurological disease Tissue Consortium (NNTC), the UCLA Brain Bank Program, various multi-center AIDS cohort study (MACS) groups, the UCSF AIDS Specimen Bank (ASB) and the Hawaiian AIDS natural history cohort study. Examples of the second type, where specimens have been transferred to the ACSR include: the San Francisco gay men's HHV-8 natural history cohort, and the US Department of Defence Thailand vaccine trial serum specimen bank.

### The ACSR approach to the changing epidemic

The USA alone has about 950,000 people living with HIV infection (about a quarter of whom are not aware of their infection). Prospective and longitudinal samples across the time-line of the changes in the epidemic are critical to understanding the epidemic as it evolves. Collection of such specimens has been a goal of the ACSR. The ACSR is taking into consideration the evolution of HIV-associated cancers (e.g., the higher rate of head and neck cancer in HIV patients in Africa) by establishing pertinent collaborations. Through collaboration, the ACSR is reaching out to heavily HIV/AIDS affected areas, such as Africa, Brazil, Russia, and Thailand to acquire relevant specimens.

## Conclusion

The ACSR makes available large numbers of HIV/AIDS-related malignancy specimens for funded, approved researchers at no fee. Scientific discovery has been advanced by this unique program through promotion of interest in and access to HIV/AIDS human tissues for primary and translational research. Investigators using ACSR specimens have shared their discoveries with the scientific community through publications in notable scientific journals and at meetings. Investigators are encouraged to browse the ACSR Internet site or contact the ACSR for specimens to enhance their own scientific initiatives.

## Competing interests

The author(s) declare that they have no competing interests.

## Authors' contributions

MSM wrote the initial draft of the manuscript. LWA had primary responsibility for rewriting, reviewing, incorporating comments and editing the manuscript. JMO also edited the manuscript. All authors provided comments of various drafts, participated in direction setting discussions and reviews and have read and approved the final version.

## Appendix I – Articles using ACSR resources

1. Kaplan LD, Shiramizu B, Herndier B, Hahan J, Meeker TC, Ng V, Volberding PA, and McGrath MS. Influence of molecular characteristics on clinical outcome in HIV-associated non-Hodgkin's lymphoma: Identification of a subgroup with favorable clinical outcome. Blood 85:1727–1735; 1995.

2. Renne R, Zhong W, Herndier B, McGrath MS, Abbey NW, Kedes D, Ganem D. Lytic growth of Kaposi's sarcoma – associated herpesvirus (human herpesvirus 8) in B cell lymphoma cells in culture. Nature Med. 2:342–346;1996.

3. Przybylski GK, Goldman J, Ng VL, McGrath MS, Herndier BG, Schenkein DP, Monroe JG, Silberstein LE. Evidence for early B-cell activation preceding the development of Epstein-Barr virus-negative acquired immunodeficiency syndrome-related lymphoma. Blood. 1996 Dec 15;88(12):4620–9.

4. Staskus KA, Zhong W, Gebhard K, Herndier B, Wang H, Renne R, Beneke J, Pudney J, Anderson DJ, Ganem D, Haase AT. Kaposi's sarcoma-associated herpesvirus gene expression in endothelial (spindle) tumor cells. J Virol. 1997 Jan;71(1):715–9.

5. Ng VL, Hurt MH, Herndier BG, Fry KE, McGrath MS. VH gene use by HIV type 1-associated lymphoproliferations. AIDS Res Hum Retroviruses. 1997 Jan 20;13(2):135–49.

6. Orenstein JM, Alkan S, Blauvelt A, Jeang KT, Weinstein MD, Ganem D, Herndier B. Visualization of human herpesvirus type 8 in Kaposi's sarcoma by light and transmission electron microscopy. AIDS. 1997 Apr;11(5):F35–45.

7. Holly EA, Lele C, Bracci P. Non-Hodgkin's lymphoma in homosexual men in the San Francisco Bay Area: occupational, chemical, and environmental exposures. J Acquir Immune Defic Syndr Hum Retrovirol. 1997 Jul 1;15(3):223–31.

8. Picchio GR, Sabbe RE, Gulizia RJ, McGrath M, Herndier BG, Mosier DE. The KSHV/HHV8-infected BCBL-1 lymphoma line causes tumors in SCID mice but fails to transmit virus to a human peripheral blood mononuclear cell graft. Virology. 1997 Nov 10;238(1):22–9.

9. Mallery SR, Landwehr DJ, Ness GM, Clark YM, Hohl CM. Thiol redox modulation of tumor necrosis factor-alpha responsiveness in cultured AIDS-related Kaposi's sarcoma cells. J Cell Biochem. 1998 Mar 1;68(3):339–54.

10. Palefsky JM, Holly EA, Hogeboom CJ, Ralston ML, DaCosta MM, Botts R, Berry JM, Jay N, Darragh TM. Virologic, immunologic, and clinical parameters in the incidence and progression of anal squamous intraepithelial lesions in HIV-positive and HIV-negative homosexual men. J Acquir Immune Defic Syndr Hum Retrovirol. 1998 Apr 1;17(4):314–9.

11. Wistuba II, Behrens C, Milchgrub S, Virmani AK, Jagirdar J, Thomas B, Ioachim HL, Litzky LA, Brambilla EM, Minna JD, Gazdar AF. Comparison of molecular changes in lung cancers in HIV-positive and HIV-indeterminate subjects. JAMA. 1998 May 20;279(19):1554–9.

12. Li M, MacKey J, Czajak SC, Desrosiers RC, Lackner AA, Jung JU. Identification and characterization of Kaposi's sarcoma-associated herpesvirus K8.1 virion glycoprotein. J Virol. 1999 Feb;73(2):1341–9.

13. Orenstein JM, Wahl SM. The macrophage origin of the HIV-expressing multinucleated giant cells in hyperplastic tonsils and adenoids. Ultrastruct Pathol. 1999 Mar-Apr;23(2):79–91.

14. Mallery SR, Clark YM, Ness GM, Minshawi OM, Pei P, Hohl CM. Thiol redox modulation of doxorubicin mediated cytotoxicity in cultured AIDS-related Kaposi's sarcoma cells. J Cell Biochem. 1999 May 1;73(2):259–77.

15. Zong JC, Ciufo DM, Alcendor DJ, Wan X, Nicholas J, Browning PJ, Rady PL, Tyring SK, Orenstein JM, Rabkin CS, Su IJ, Powell KF, Croxson M, Foreman KE, Nickoloff BJ, Alkan S, Hayward GS. High-level variability in the ORF-K1 membrane protein gene at the left end of the Kaposi's sarcoma-associated herpesvirus genome defines four major virus subtypes and multiple variants or clades in different human populations. J Virol. 1999 May;73(5):4156–70.

16. Poole LJ, Zong JC, Ciufo DM, Alcendor DJ, Cannon JS, Ambinder R, Orenstein JM, Reitz MS, Hayward GS. Comparison of genetic variability at multiple loci across the genomes of the major subtypes of Kaposi's sarcoma-associated herpesvirus reveals evidence for recombination and for two distinct types of open reading frame K15 alleles at the right-hand end. J Virol. 1999 Aug;73(8):6646–60.

17. Holly EA, Lele C, Bracci PM, McGrath MS. Case-control study of non-Hodgkin's lymphoma among women and heterosexual men in the San Francisco Bay Area, California. Am J Epidemiol. 1999 Aug 15;150(4):375–89.

18. Aoki Y, Tosato G, Nambu Y, Iwamoto A, Yarchoan R. Detection of vascular endothelial growth factor in AIDS-related primary effusion lymphomas. Blood. 2000 Feb 1;95(3):1109–10.

19. Martin JN, Amad Z, Cossen C, Lam PK, Kedes DH, Page-Shafer KA, Osmond DH, Forghani B. Use of epidemiologically well-defined subjects and existing immunofluorescence assays to calibrate a new enzyme immunoassay for human herpesvirus 8 antibodies. J Clin Microbiol. 2000 Feb;38(2):696–701.

20. McGrath MS, Shiramizu B, Herndier BG. Clonal HIV in the pathogenesis of AIDS-related lymphoma. In Infectious Causes of Cancer Targets for Intervention. Edited by Goedert JJ. New Jersey: Humana Press; 2000:231–242.

21. Edelman DC, Ketema F, Saville RD, Herman J, Sill AM, Gill PS, Blattner WA, Constantine NT. Specifics on the refinement and application of two serological assays for the detection of antibodies to HHV-8. J Clin Virol. 2000 May;16(3):225–37.

22. Aoki Y, Yarchoan R, Braun J, Iwamoto A, Tosato G. Viral and cellular cytokines in AIDS-related malignant lymphomatous effusions. Blood. 2000 Aug 15;96(4):1599–601.

23. Mallery SR, Pei P, Kang J, Ness GM, Ortiz R, Touhalisky JE, Schwendeman SP. Controlled-release of doxorubicin from poly(lactide-co-glycolide) microspheres significantly enhances cytotoxicity against cultured AIDS-related Kaposi's sarcoma cells. Anticancer Res. 2000 Sep-Oct;20(5A):2817–25.

24. Mallery SR, Pei P, Kang J, Zhu G, Ness GM, Schwendeman SP. Sustained angiogenesis enables in vivo transplantation of mucocutaneous derived AIDS-related Kaposi's sarcoma cells in murine hosts. Carcinogenesis. 2000 Sep;21(9):1647–53.

25. Napolitano LA, Grant RM, Deeks SG, Schmidt D, De Rosa SC, Herzenberg LA, Herndier BG, Andersson J, McCune JM. Increased production of IL-7 accompanies HIV-1-mediated T-cell depletion: implications for T-cell homeostasis. Nat Med. 2001 Jan;7(1):73–9.

26. Vilchez RA, Lednicky JA, Halvorson SJ, White ZS, Kozinetz CA, Butel JS. Detection of polyomavirus simian virus 40 tumor antigen DNA in AIDS-related systemic non-Hodgkin lymphoma. J Acquir Immune Defic Syndr. 2002 Feb 1;29(2):109–16.

27. McGrath MS, Kahn JO, Herndier BG. Development of WF10, a novel macrophage-regulating agent. Curr Opin Investig Drugs. 2002 Mar;3(3):365–73.

28. Shivapurkar N, Harada K, Reddy J, Scheuermann RH, Xu Y, McKenna RW, Milchgrub S, Kroft SH, Feng Z, Gazdar A. Presence of simian virus 40 DNA sequences in human lymphomas. The Lancet, March 9, 2002; 359:851–852.

29. Vilchez RA, Madden CR, Kozinetz CA, Halvorson SJ, White ZS, Jorgensen JL, Finch CJ, Butel JS. Association between simian virus 40 and non-Hodgkin lymphoma. Lancet. 2002 Mar 9;359(9309):817–23.

30. Lore K, Sonnerborg A, Brostrom C, Goh LE, Perrin L, McDade H, Stellbrink HJ, Gazzard B, Weber R, Napolitano LA, van Kooyk Y, Andersson J.Accumulation of DC-SIGN+CD40+ dendritic cells with reduced CD80 and CD86 expression in lymphoid tissue during acute HIV-1 infection. AIDS. 2002 Mar 29;16(5):683–92.

31. Gascon RL, Narvaez AB, Zhang R, Kahn JO, Hecht FM, Herndier BG, McGrath MS. Increased HLA-DR expression on peripheral blood monocytes in subsets of subjects with primary HIV infection is associated with elevated CD4 T-cell apoptosis and CD4 T-cell depletion. J Acquir Immune Defic Syndr. 2002 Jun 1;30(2):146–53.

32. Rubenstein JL, Combs D, Rosenberg J, Levy A, McDermott M, Damon L, Ignoffo R, Aldape K, Shen A, Lee D, Grillo-Lopez A, Shuman MA. Rituximab therapy for CNS lymphomas: targeting the leptomeningeal compartment. Blood. 2003 Jan 15;101(2):466–8. Epub 2002 Sep 5.

33. Zenger E, Abbey NW, Weinstein MD, Kapp L, Reis J, Gofman I, Millward C, Gascon R, Elbaggari A, Herndier BG, McGrath MS. Injection of human primary effusion lymphoma cells or associated macrophages into severe combined immunodeficient mice causes murine lymphomas. Cancer Res. 2002 Oct 1;62(19):5536–42.

34. Spiller OB, Robinson M, O'Donnell E, Milligan S, Morgan BP, Davison AJ, Blackbourn DJ. Complement regulation by Kaposi's sarcoma-associated herpesvirus ORF4 protein. J Virol. 2003 Jan;77(1):592–9.

35. Romer DJ, Suster S. Use of virtual microscopy for didactic live-audience presentation in anatomic pathology. Ann Diagn Pathol. 2003 Feb;7(1):67–72.

36. Dittmer DP. Transcription profile of Kaposi's sarcoma-associated herpesvirus in primary Kaposi's sarcoma lesions as determined by real-time PCR arrays. Cancer Res. 2003 May 1;63(9):2010–5.

37. Mallery SR, Morse MA, Wilson RF, Pei P, Ness GM, Bradburn JE, Renner RJ, Schuller DE, Robertson FM. AIDS-related Kaposi's sarcoma cells rapidly internalize endostatin, which co-localizes to tropomysin microfilaments and inhibits cytokine-mediated migration and invasion. J Cell Biochem. 2003 May 1;89(1):133–43.

38. Romer DJ, Yearsley KH, Ayers LW. Using a modified standard microscope to generate virtual slides. Anat Rec B New Anat. 2003 May;272(1):91–7.

39. Haque M, Davis DA, Wang V, Widmer I, Yarchoan R. Kaposi's sarcoma-associated herpesvirus (human herpesvirus 8) contains hypoxia response elements: relevance to lytic induction by hypoxia. J Virol. 2003 Jun;77(12):6761–8.

40. Mack KD, Jin X, Yu S, Wei R, Kapp L, Green C, Herndier B, Abbey NW, Elbaggari A, Liu Y, McGrath MS.HIV insertions within and proximal to host cell genes are a common finding in tissues containing high levels of HIV DNA and macrophage-associated p24 antigen expression. J Acquir Immune Defic Syndr. 2003 Jul 1;33(3):308–20.

41. Chaves AA, Mihm MJ, Schanbacher BL, Basuray A, Liu C, Ayers LW, Bauer JA. Cardiomyopathy in a murine model of AIDS: evidence of reactive nitrogen species and corroboration in human HIV/AIDS cardiac tissues. Cardiovasc Res. 2003 Oct 15;60(1):108–18.

42. Mallery SR, Pei P, Landwehr DJ, Clark CM, Bradburn JE, Ness GM, Robertson FM. Implications for oxidative and nitrative stress in the pathogenesis of AIDS-related Kaposi's sarcoma. Carcinogenesis. 2004 Apr;25(4):597–603. Epub 2003 Dec 4.

43. Killebrew DA, Troelstrup D, Shiramizu B. Preferential HIV-1 integration sites in macrophages and HIV-associated malignancies. Cell Mol Biol (Noisy-le-grand). 2004;50 Online Pub:OL581–9.

44. McAllister SC, Hansen SG, Ruhl RA, Raggo CM, DeFilippis VR, Greenspan D, Fruh K, Moses AV. Kaposi sarcoma-associated herpesvirus (KSHV) induces heme oxygenase-1 expression and activity in KSHV-infected endothelial cells. Blood. 2004 May 1;103(9):3465–73. Epub 2004 Jan 15.

45. Milligan S, Robinson M, O'Donnell E, Blackbourn DJ. Inflammatory cytokines inhibit Kaposi's sarcoma-associated herpesvirus lytic gene transcription in in vitro-infected endothelial cells. J Virol. 2004 Mar;78(5):2591–6.

46. Deeks SG, Kitchen CM, Liu L, Guo H, Gascon R, Narvaez AB, Hunt P, Martin JN, Kahn JO, Levy J, McGrath MS, Hecht FM. Immune activation set point during early HIV infection predicts subsequent CD4+ T-cell changes independent of viral load. Blood. 2004 Aug 15;104(4):942–7. Epub 2004 Apr 29.

47. Giese T, McGrath MS, Stumm S, Schempp H, Elstner E, Meuer SC. Differential effects on innate versus adaptive immune responses by WF10. Cell Immunol. 2004 Jun;229(2):149–58.

48. Wang HW, Trotter MW, Lagos D, Bourboulia D, Henderson S, Makinen T, Elliman S, Flanagan AM, Alitalo K, Boshoff C. Kaposi sarcoma herpesvirus-induced cellular reprogramming contributes to the lymphatic endothelial gene expression in Kaposi sarcoma. Nat Genet. 2004 Jul;36(7):687–93. Epub 2004 Jun 27.

49. Zhang R, Gascon R, Miller RG, Gelinas DF, Mass J, Hadlock K, Jin X, Reis J, Narvaez A, McGrath MS. Evidence for systemic immune system alterations in sporadic amyotrophic lateral sclerosis (sALS). J Neuroimmunol. 2005 Feb;159(1–2):215–24. Epub 2004 Nov 26.

50. Marchiò S., Alfano M., Primo L., Gramaglia D., Butini L., Gennero L., De Vivo E., Arap W., Giacca M., Pasqualini R., Bussolino F. Cell Surface-associated Tat modulates HIV-1 infection and spreading through a specific interaction with gp120 viral envelope protein. Blood 2005 April 1; 105(7):2802–11

51. Kurokawa M, Ghosh SK, Ramos JC, Mian AM, Toomey NL, Cabral L, Whitby D, Barber GN, Dittmer DP, Harrington WJ Jr. Azidothymidine inhibits NF-kappaB and induces Epstein-Barr virus gene expression in Burkitt lymphoma. Blood. 2005 Jul 1;106(1):235–40. Epub 2005 Mar 24.

52. Orem J, Otieno MW, Banara C, Katongoli-Mbidde E, Johnson JL, Ayers L, Ghannoum M, Fu P, Feigal EG, Black J, Whalen G, Ledeman M, Remick SC: Capacity Building for the Clinical Investigation of AIDS Maligancy in East Africa. Cancer Detection and Prevention. 2005 April 26;29(2):133–4.

53. Nohle DG, Ayers LW. The tissue microarray data exchange specification: a document type definition to validate and enhance XML data. BMC Med Inform Decis Mak. 2005 May 4;5(1):12.

54. Baliga RS, Chaves AA, Jing L, Ayers LW, Bauer JA. AIDS-related vasculopathy: evidence for oxidative and inflammatory pathways in murine and human AIDS. Am J Physiol Heart Circ Physiol. 2005 Oct;289(4):H1373–H1380. Epub 2005 May 27.

55. Nohle DG, Hackman BA, Ayers LW. The tissue micro-array data exchange specification: a web based experience browsing imported data. BMC Med Inform Decis Mak. 2005 Aug 8;5:25.

56. Salemi M, Lamer SL, Yu S, de Oliveira T, Fitch WM, McGrath MS. Phylodynamic analysis of Human Immunodeficiency Virus Type 1 in Distinct Brain Compartments Provides a Model for the Neuropathogenesis of AIDS. J Virol, Sept, 2005. vol 79 No 17, p. 11343–11352.

57. Curry CL, Reed LL, Golde TE, Miele L, Nickoloff BJ, and Foreman KE. Gamma secretase inhibitor blocks Notch activation and induces apoptosis in Kaposi's sarcoma tumor cells. Oncogene 2005 Sep 22;24:6333–44.

58. Krathwohl MD, Schacker TW, and Anderson JL. Abnormal Presence of Semimature Dendritic Cells That Induce Regulatory T Cells in HIV-Infected Subjects. J Infect Dis 2006 Feb 15; 193:494–504

59. Chaves AA, Baliga RS, Mihm MJ, Schanbacher BL, Basuray A, Liu C, Cook AC, Ayers LW, Baurer JA. Bacterial Lipopolysaccharide Enhances Cardiac Dysfunction but Not Retroviral Replication in Murine AIDS: Roles of Macrophage Infiltration and Toll-Like Receptor 4 Expression. Am J Pathol 2006 Mar;168(3):727–735

60. Wang L, Dittmer DP, Tomlinson CC, Fakhari FD, Damania B. Immortalization of primary endothelial cells by the K1 protein of Kaposi's sarcoma-associated herpesvirus. Cancer Res. 2006 Apr 1;66(7):3658–66

61. Zhang R, Gascon R, Miller RG, Gelinas DF, Mass J, Lancero M, Narvaez A, McGrath MS. MCP-1 chemokine receptor CCR2 is decreased on circulating monocytes in sporadic amyotrophic lateral sclerosis (sALS). J Neuroimmunol. 2006 Oct;179(1–2):87–93. Epub 2006 Jul 20.

## Appendix II – Presentation abstracts using ACSR resources

1. Mack K, Wei R, Shiramizu B, Herndier B, Elbaggari A, Gascon R, Hurt M, McGrath MS. "Sequential Pathogenesis" of AIDS malignancies: Clonal insertion of HIV into macrophages may serve as an early neoplastic event. Keystone Symposia on Molecular and Cellular Biology Park City, UT, March 13–19, 1998

2. King MA, Yip D, Neal DE, Wewers M, Pacht ER, Gadek J, Diaz PT, Ayers LW. Occult pulmonary hemorrhage in HIV-positive individuals without AIDS correlates with CT evidence of emphysema. Am J Respir Crit Care Med 1998:157:A1784.

3. Flickinger M, McGrath M, Silver S, Orenstein J, Miles S, Ayers LW. AIDS Malignancy Bank: a source for tissue and biological fluids of HIV-related malignancies. In Proceedings of the Second National AIDS Malignancy Conference. Bethesda, Maryland, USA. April 6–8, 1998. J Acquir Immune Defic Syndr. 1999 Aug 1;21 Suppl 1:S1–86.

4. Herndier B, Wei R, Abbey N, Weinstein M, Mack K, Shiramizu B, McGrath MS. Kaposi's sarcoma: a multicellular, mulitviral process initiated by retroviral insertional mutagenesis?. In Proceedings of the Second National AIDS Malignancy Conference. Bethesda, Maryland, USA. April 6–8, 1998. J Acquir Immune Defic Syndr. 1999 Aug 1;21 Suppl 1:S1–86.

5. Mack K, Wei R, Shiramizu B, Herndier B, Elbaggari A, Gascon R, Hurt M, McGrath MS. Evidence for HIV mediated cis-activation of the c-fes protooncogene in a subset of AIDS associated lymphomas. In Proceedings of the Second National AIDS Malignancy Conference. Bethesda, Maryland, USA. April 6–8, 1998. J Acquir Immune Defic Syndr. 1999 Aug 1;21 Suppl 1:S1–86.

6. Mallery SR, Clark YM, Ness GM, Ayers LW and CM Hohl. AIDS-Kaposi's Sarcoma Demonstrate Increased Susceptibility to a Redox Cycling Drug. In Proceedings of the Second National AIDS Malignancy Conference. Bethesda, Maryland, USA. April 6–8, 1998. J Acquir Immune Defic Syndr. 1999 Aug 1;21 Suppl 1:S1–86.

7. Flickinger M, McGrath M, Silver S, Orenstein J, Miles S, Ayers LW. AIDs malignancy bank: a source for tissue and biological fluids of HIV-related malignancies. 12th World AIDS Conference Geneva, Switzerland, June 28 – July 3, 1998 [Conference WebSite]

8. McGrath MS, Shiramizu B, McGuire D, Pulliam L, Herndier B. HIV-associated neoplastic dementia: A model implicating a role for clonal macrophages. In Proceedings of the Second National AIDS Malignancy Conference. Bethesda, Maryland, USA. April 6–8, 1998. J Acquir Immune Defic Syndr. 1999 Aug 1;21 Suppl 1:S1–86.

9. Ayers LW, McGrath MS, Silver S, Miles S, Axiotis C. The AIDS and Cancer Specimen Bank (ACSB), A NCI Tissue and Biological Fluids Bank of HIV Infected Tissues. In The 3rd National AIDS Malignancy Conference. Bethesda, Maryland, USA. May 26–27, 1999. Abstracts. J Acquir Immune Defic Syndr. 1999 May 1;21(1):A1–41.

10. Flickinger M, McGrath M, Silver S, Orenstein J, Miles S, Ayers LW, Axiotis C. Tissue and biological fluids banks of HIV-related malignancies. In The 3rd National AIDS Malignancy Conference. Bethesda, Maryland, USA. May 26–27, 1999. Abstracts. J Acquir Immune Defic Syndr. 1999 May 1;21(1):A1–41.

11. Parris L, Ayers LW. Iron Loading of Alveolar Macrophages in HIV Positive Patients. The MRI Science Symposium, Medical/Science Research Initiative, (MRI), Office of Minority Affairs Columbus, OH, August 1999

12. Abbey NW, McGrath MS, Herndier BG. C-fes proto-oncogene expression in HIV and non-HIV lymphoid tissues. Seventh Conference on Retroviruses and Opportunistic Infections San Francisco, CA, January 30 – February 2, 2000

13. Mack KD, Wei R, Herndier B, Shiramizu B, Abbey N, Gascon R, Elbaggari A, Hurt M, Earnst T, McGrath MS. HIV implicated in the cis-activation of c-fes in a subset of HIV associated lymphomas. Seventh Conference on Retroviruses and Opportunistic Infections San Francisco, CA, January 30 – February 2, 2000

14. Davis, AT, Mosunjac MB. Cervical dysplasia in women infected with human immunodeficiency virus {HIV): a correlation with HIV viral load. United States and Canadian Association of Pathology meeting New Orleans, LA, March 2000

15. Davis, AT, Walliang R, Mosunjac MI, Mosunjac MB. Human immunodeficiency virus (HIV)-associated premalignant and malignant lesions by gender in a large inner-city hospital: a retrospective study. United States and Canadian Academy of Pathology Annual Meeting New Orleans, LA, March 25–31, 2000

16. Abbey NW, Silver S, Orenstein JM, Miles S, Ayers L, Axiotis C, McGrath MS. The AIDS and Cancer Specimen Bank. In The 4th International AIDS Malignancy Conference. Bethesda, Maryland, USA. May 16–18, 2000. Abstracts. J Acquir Immune Defic Syndr. 2000 Mar 1;23(3):A1–37.

17. Ayers LW, and The Midregion ACSB Consortium. The Ohio State University, Emory University, Rush University, University of Texas, Southwestern and Vanderbilt University: Application of the Tissue Microarray (TMA) method by the Midregion AIDS and Cancer Specimen Bank (ACSB) to prepare study sets from HIV infected and control tissues. In The 4th International AIDS Malignancy Conference. Bethesda, Maryland, USA. May 16–18, 2000. Abstracts. J Acquir Immune Defic Syndr. 2000 Mar 1;23(3):A1–37.

18. McGrath MS, Elbaggari A, Gascon R, Herndier B, Meuer S, Giese T, Zenger E. Quantitation of AIDS lymphoma associated macrophage RNA. implications for "sequential pathogenesis" of extranodal lymphoma. In The 4th International AIDS Malignancy Conference. Bethesda, Maryland, USA. May 16–18, 2000. Abstracts. J Acquir Immune Defic Syndr. 2000 Mar 1;23(3):A1–37.

19. Ayers LW, Romer D, Yi H, The Midregion AIDS and Cancer Specimen Bank (ACSB). Cancer Research Data from Tissue Microarrays (TMA) and the Associated Patient Demographic and Clinical Information Facilitated by a Graphical Microsoft Access Database. Cancer research data from tissue microarrays (TMA) and the associated patient demographic and clinical information facilitated by a graphical Microsoft Access database. Third Annual Comprehensive Cancer Center Scientific Meeting Dublin, OH, January 24, 2001

20. Ayers LW, Romer D, Yi H, and The Midregion AIDS and Cancer Specimen Bank (ACSB), and Division of Informatics, Department of Pathology, The Ohio State University, Columbus, OH 43210. AIDS-Associated Malignant Tissue Research using Tissue Micro-Arrays (TMAs) Facilitated by a Graphical Microsoft Access Database. Fifth International AIDS Malignancy Conference Bethesda, MD, April 23–26, 2001 [program only]

21. Garcia D, McGrath MS, Silver S, Miles S, Ayers L, Axiotis C. AIDS and Cancer Specimen Bank. Fifth International AIDS Malignancy Conference Bethesda, Maryland, 26, April 23–25, 2001 [program only]

22. Ayers L, Romer D. Development of a Tissue Array Data System (TADS) for AIDS and Cancer Specimen Bank (ACSB). In Advancing Pathology Informatics, Imaging, and the Internet (APIII 2001). Arch Pathol Lab Med. 2002 Jul;126(7):791–792.

23. Romer D, Ayers L. Adding a Robotic Stage to the Desktop Microscope and Creating Virtual Slides with Image Pro Plus. In Advancing Pathology Informatics, Imaging, and the Internet (APIII 2001). Arch Pathol Lab Med. 2002 Jul;126(7):792.

24. Garcia DL, McGrath MS, Silver S, Orenstein J, Ayers L. AIDS and Cancer Specimen Bank. The XIV International AIDS Conference, 2002 Abstract no. C11034.

25. Ayers LW, Romer D, Yi H. The Midregion AIDS and Cancer Specimen Bank (ACSB). A Tissue Array Database System (TADS) for the AIDS Cancer and Specimen Bank (ACSB) Developed to Manage Numerical and Digital Image Cancer Research Data From Tissue Microarrays (TMAs). The Fourth Annual Comprehensive Cancer Center Scientific Meeting Dublin, OH, January 11, 2002

26. Mihm M, Schanbacher BL, Basuray A, Lui C, Amann DM, Ayers LW, John Anthony Bauer, JA. Protein Oxidation and Immune Cell Trafficking in Human AIDS-Related Cardiomyopathy. The Ohio State University Medical Center Graduate and Postgraduate Research Day April 4, 2002

27. Ayers LW, D. Romer, H Yi. The Midregion AIDS and Cancer Specimen Bank (ACSB). A Tissue Array Management System (TAMS) for the AIDS Cancer and Specimen Bank (BANK) Developed to Manage Numerical and Digital Image Cancer Research Data From Tissue Microarrays (TMAs). Sixth International Conference on Malignancies in AIDS and Other Immunodeficiencies: Basic, Epidemiologic and Clinical Research Bethesda, MD, April 22–24, 2002 [program only]

28. Garcia DL, Ayers LW, Axiotis C, Orenstein J, Miles S, Silver S, McGrath MS. ACSB CODCC, AIDS and Cancer Specimen Bank: A Resource for Your Research. Sixth International Conference on Malignancies in AIDS and Other Immunodeficiencies: Basic, Epidemiologic and Clinical Research Bethesda, MD, April 22–24, 2002 [program only]

29. McGrath MS, Jin X, Yu S, Wei R, Kapp L, Green C, Abbey NW, Elbaggari A, Liu Y, Herndier B, Mack KD. HIV insertions within and proximal to host cell genes are a common finding in tissues containing high levels of HIV DNA and macrophage associated P24 antigen expression. Fifth International Workshop on HIV, Cells of Macrophage/Dendritic Lineage and Other Reservoirs Rome, Italy, 15, October 13–15, 2002.

30. Hadlock KG, Miller RG, Gelinas D, Jin X, Reis J, Mass J, McGrath MS. Elevated reactivity to HML-2/HERV-K but not other endogenous retroviruses in ALS. 55th Annual Meeting of the American Academy of Neurology Honolulu, HI, March 29-April 5, 2003

31. McGrath MS, Gascon RL, Miller RG, Gelinas D, Yu S, Zhang RZ, Frydman B, Marton L. Pathogenesis of ALS: Removal of abnormal macrophages (MO) and source of pathogenic factor production from ALS blood in vitro with a novel polyamine analogue, SL-11047. 55th Annual Meeting of the American Academy of Neurology Honolulu, HI, March 29-April 5, 2003

32. Ayers LW, Mihm MJ, Hackman B, Bauer JA, Midregion AIDS and Cancer Specimen Resource (ACSR). AIDS Patients have High Levels of Protein Nitration within Actively HIV Infected Cells. Does this Cause Organ Malfunction and Cancer? Abstracts from the Seventh International Conference on Malignancies in AIDS and Other Immunodeficiencies: Basic, Epidemiologic and Clinical Research, Natcher Conference Center Bethesda, MD, April 28–29, 2003. 2003:86.

33. Silver S, Ayers L, Orenstein J, McGrath M. Aids and Cancer Specimen Resource: a Resource for Your Research. In Abstracts of the ISBER Annual Meeting: Biodiversity and International Regulations. May 4–7, 2003, Philadelphia, Pennsylvania, USA. Biotech Histochem. 2003 Jun-Aug;78(3–4):207–28.

34. Nohle DG, Hackman BA, Ayers LW. Web-based Virtual Tissue Micro-array Slides with clinical Data F or Researchers in HTML, Excel and API Standard XML Data Exchange Specification Formats Produced with Microsoft Office^® ^Applications. In Advancing Practice, Instruction, and Innovation Through Informatics (APIII 2003): Scientific Session and E-Poster Abstracts. Arch Pathol Lab Med. 2004 Oct;128(10):1089–1123.

35. Bachowski GJ, Birdsong GG, Lau SK, Tadros TS, Oprea GM. Evolution of High Grade Squamous Intraepithelial Lesion (HSIL) in HIV-Positive Women compared to the General Population: 5 year Follow-Up. United States and Canadian Academy of Pathology Meeting Vancouver, Canada. March 17–19, 2004

36. Yu S, Lamers S, Jin X, Reis J, McGrath MS. Restricted HIV genomic variability in AIDS dementia and lymphoma. Keystone Symposia, Molecular mechanisms of HIV Pathogenesis Whistler, British Columbia, April 12–18, 2004 [program only]

37. Ayers LW, Jin X, Bauer JA, McGrath M. HIV Integration Sites Found in Cardiac Myocyte Nuclei and Adjacent PCNA Positive Macrophage Nuclei from AIDS Cardiomopathy Heart Tissue. Abstracts from the 8th International Conference on Malignancies in AIDS and Other Immunodeficiencies: Basic, Epidemiologic and Clinical Research Bethesda, MD, April 29–30, 2004. 2004:74.

38. Lamers S, Yu S, McGrath MS. Genetic analysis of HIV-1 from site-specific brain tissue: Does compartmentalization occur within the brain? 11th International Workshop, HIV Dynamics and Evolution Stockholm, Sweden, April 29-May 4, 2004

39. Nohle DG, Hackman B, Ayers L. Tissue Micro-Array Data Using TMA Standard and Public Tool Linked Legend and Clinical Details at ACSR.Mid-Region.org. Tissue Micro-Array Data Using TMA Standard and Public Tool Linked Legend and Clinical Details at acsr.mid-region.org. Abstracts from the 8th International Conference on Malignancies in AIDS and Other Immunodeficiencies: Basic, Epidemiologic and Clinical Research, Natcher Conference Center Bethesda, MD, April 29–30, 2004. 2004:75.

40. McGrath MS, Garcia D, Ayers L, Silver S, Orenstein J, Lamers S. AIDS and cancer specimen resource: A resource for HIV reservoir research. The XV International AIDS Conference, 2004 Abstract no. A10525.

41. McGrath MS, Yu S, Jin X, Reis J, Lamers S. Restricted HIV genomic variability in AIDS dementia and lymphoma. The XV International AIDS Conference, 2004 Abstract no. ThOrA1399.

42. Silver S, Ayers L, Orenstein J, McGrath M. AIDS and Cancer Specimen Resource (ASCR): A Resource for Your Research. International Society of Biological and Environmental Repositories, Perugia, Italy, October 2004

43. Lee Z, Muzic R, Gagon D. Dynamic PET for Animal Model of HCC. Presented at Annual Meeting of Biomedical Engineering Society, October 2004

44. Silver S, Ayers L, Orenstein J, McGrath M. The AIDS and Cancer Specimen Resource (ACSR) Model for Cancer Research Facilitation. International Society of Biological and Environmental Repositories, Bellevue, WA, May 2005

45. Nohle DG, Hackman BA, Ayers LW. A Document Type Definition for the API standard tissue micro-array XML data exchange specification, In Advancing Practice, Instruction, and Innovation Through Informatics (APIII 2004): Scientific Session and E-Poster Abstracts. Arch Pathol Lab Med. 2005 Jun;129(6):811–834.

46. Lamers SL, Salemi M, McGrath MS. HIV-1 envelope domains derived from AIDS related lymphoma tissues display unique substitution patterns as compared to published envelope domains. The 3rd IAS Conference on HIV Pathogenesis and Treatment, 2005 Abstract no. MoPe14.2B17.

47. McGrath MS, Lamers SL, Salemi M. HIV sequence evolution in the brain: Distinctly different evolutionary pathways between HAD and primary CNS lymphoma associated strains of HIV. The 3rd IAS Conference on HIV Pathogenesis and Treatment, 2005 Abstract no. WePe8.13B04.

48. Nohle DG, Hackman BA, Ayers LW. Adding style to the API Tissue Micro-array Data Exchange Specification, In Advancing Practice, Instruction, and Innovation Through Informatics (APIII 2005): Scientific Session and E-Poster Abstracts. Arch Pathol Lab Med.

49. McGrath MS, Salemi M, Lamers SL. Genetic Analysis of HIV in AIDS Malignancies. Oral presentation by Michael S. McGrath, MD, PhD at the 2005 International Meeting of the Institute of Human Virology, Baltimore, MD, Aug 29-Sep 2, 2005.

50. Dittmer DP, Staudt MR, Fakhari FD, Hilscher C, Sin S, Chaput A. Regulation of KSHV Latent gene expression. Abstracts from the 9th International Conference on Malignancies in AIDS and Other Acquired Immunodeficiencies: Basic, Epidemiologic and Clinical Research, Marriott Bethesda North Hotel & Conference Center, North Bethesda, MD, September 26–27, 2005. 

51. Levine AL, Seaberg EC, Hessol NA, Preston-Martin S, Li M, Silver S, Cohen M, Anastos K, Minkoff H, Black J, Watts DH. HIV is not a risk factor for lung cancer in women: data from the women's interagency HIV study (WIHS). Abstracts from the 9th International Conference on Malignancies in AIDS and Other Acquired Immunodeficiencies: Basic, Epidemiologic and Clinical Research, Marriott Bethesda North Hotel & Conference Center, North Bethesda, MD, September 26–27, 2005. 

52. Mbulaiteye SM, Katabira ET, Wabinga H, Parkin DM, Virgo P, Ochai R, Workneh M, Coutinho A, Engels EA. Spectrum of cancers among HIV-infected persons in Africa: the Uganda AIDS-cancer registry match study. Abstracts from the 9th International Conference on Malignancies in AIDS and Other Acquired Immunodeficiencies: Basic, Epidemiologic and Clinical Research, Marriott Bethesda North Hotel & Conference Center, North Bethesda, MD, September 26–27, 2005. 

53. McGrath MS, Lamers S, Salemi M. Evolutionary distinct forms of HIV in AIDS lymphoma and dementia. Abstracts from the 9th International Conference on Malignancies in AIDS and Other Acquired Immunodeficiencies: Basic, Epidemiologic and Clinical Research, Marriott Bethesda North Hotel & Conference Center, North Bethesda, MD, September 26–27, 2005. 

54. McGrath MS, Lamers SL, Salemi M. HIV infected macrophages associated with dementia (HAD) and lymphoma (ARL) show distinct patterns of evolution in vivo. Poster presentation at the 6th International Workshop on HIV, Cells of Macrophage/Dendritic Lineage & Other Reservoirs, Varenna, Italy, Oct 5–7 2005.

55. Curry CL, Reed LL, Nickoloff BJ, and Foreman KE. Targeting Notch in Kaposi's sarcoma inhibits tumorigenesis. Oral presentation at Society of Investigative Dermatology Meeting, 2004; Oral presentation at Seventh International Workshop on Kaposi's Sarcoma-Associated Herpesvirus (KSHV/HHV8) and Related Agents, 2004; Poster presentation at EMBO Notch Workshop, 2005.

56. Shikuma CM, Shiramizu B, McGrath M, Shagrun L, Chow D, Gerschenson M. HIV Infected Monocytes and Macrophages in Adipose Tissue Contribute to the Development of Lipatrophy. 7th International Workshop on Adverse Drug Reactions and Lipodystrophy in HIV, 13–16 Nov 2005 in Dublin, Ireland.

57. Huerta-Yepez S, Vega M, Gui D, Said J, Bonavida B. Analysis of YY1 and XIAP expression, proteins that regulate resistance, in AIDS-NHL tissue arrays. Blood (ASH Annual Meeting Abstracts) 2005 106: Abstract 1933 2005 Dec;106 (11).

58. Zhang RZ, Gascon R, Miller RG, Gelinas DF, Mass J, Lancero M, Narvaez A, McGrath MS. MCP-1 Chemokine Receptor CCR2 Is Absent on Circulating Monocytes in Sporadic ALS. Poster presentation at the 16th International Symposium on ALS/MND in Dublin, Ireland, Dec 8–10, 2005.

59. Do H, Hadlock KG, Miller RG, Yu S, Mass J, Zhang RZ, Gascon R, Narvaez A, Katz J, McGrath, MS. Transcriptional Program of Peripheral Blood Cells in ALS: Evidence for Systemic Immune Activation. Poster presentation at the 16th International Symposium on ALS/MND in Dublin, Ireland, Dec 8–10, 2006.

60. Jin X, Hadlock KG, Yu S, Do H, Miller R, Mass J, Katz J, Zhang RZ, Gascon R, McGrath MS. Mutational Recovery of Viral Open Reading Frames in Selected HERV-K-Related Endogenous Retro Viruses in ALS Patients. Poster presentation at the 16th International Symposium on ALS/MND in Dublin, Ireland, Dec 8–10, 2005.

61. Yu S, Hadlock KG, Miller RG, Do H, Mass J, Zhang RZ, Gascon R, Lancero M, Narvaez A, Katz J, McGrath MS. Detection of ALS by Quantitative Real Time RT-PCR of Peripheral Blood Cells. Poster presentation at the 16th International Symposium on ALS/MND in Dublin, Ireland, Dec 8–10, 2005.

62. Gruzman AL, Prasad MD, Miller RG, Lingappa V & Liu J. Identification of Biomarkers for ALS. Poster presentation at the 16th International Symposium on ALS/MND in Dublin, Ireland, Dec 8–10, 2005.

63. Marchiò S., Bagnod R., Traversa S., Fumero S., Bussolino F.: A new peptide inhibitor of HIV-1 entry. Poster presented at the congress "PepTalk", San Diego, CA. January 11th-13th, 2006.

64. McGrath MS, Galligan D, Lamers S, Salemi M. HIV Infected Macrophages Associated with Lymphoma (ARL) Show Distinct Patterns of Evolution in Vivo. Accepted for poster presentation at 13th Conference on Retroviruses and Opportunistic Infections, Denver CO, Feb 5–9, 2006.

65. Galligan D, Lamers SL, de Oliveira T, Salemi, M, McGrath MS. HIV-1 Molecular Dynamics in Multisite Lymphoma Biopsies. Accepted for poster presentation at HIV Pathogenesis Symposium, Keystone CO, Mar 27-Apr 2, 2006.

66. Lamers SL, Salemi T, de Oliveria T, Beason S, Compton R, Goodenow M, McGrath MS. A Protocol for HIV-1 env Alignments Assists in Determining the Evolutionary Dynamics of the V1, V2 and V4 Hypervariable Domains. Presented to HIV Pathogenesis Symposium, Keystone CO, Mar 27-Apr 2, 2006

67. Nohle DG, Ayers LW. Extending the API TMA data exchange specification to single specimen and cutting edge matrix assembly (CEMA). ASIP INFORMATICS, Experimental Biology 2006: Advancing the Biomedical Frontier. The Moscone Convention Center, San Francisco, CA, April 1 – 5, 2006.

68. Ayers LW, McGrath MS, Silver S, Orenstein J, Bhatia K. RNA, DNA and Molecular Targets in Archived Paraffin-embedded Tissue Samples, 1980–2005. Presented to International Society for Biological and Environmental Repositories (ISBER) 2006 Annual Meeting, Bethesda, MD. April 30-May 3, 2006.

69. Ayers LW, Hackman BA, Nohle DG. Software-Assisted Image Analysis for ACSR Tissue Microarays (TMA) Quality Control. Presented to International Society for Biological and Environmental Repositories (ISBER) 2006 Annual Meeting, Bethesda, MD. April 30-May 3, 2006.

70. Kulkarni AL, Handorf CR. Legal Issues Involving Research Utilization of Archived Formalin Fixed Paraffin Embedded Tissue. Presented to International Society for Biological and Environmental Repositories (ISBER) 2006 Annual Meeting, Bethesda, MD. April 30-May 3, 2006.

71. Ayers LW, McGrath MS, Silver S. Orenstein J., Bhatia K. The AIDS and Cancer Specimen Resource (ACSR) provides Kaposi's sarcoma (KS) and non-Hodgkin's lymphoma (NHL) specimens for translational research. Presented at the 9th Annual Workshop on KS Associated Herpesvirus & Related Viruses, Cape Cod, MA. July 12–15, 2006.

72. Carroll PA. Latent KSHV Infection of Endothelial Cells Activates Hypoxia-Induced Factors. Accepted for oral presentation (Abstract 36) at 9th Annual KSHV Workshop, Hyannis, MA. July 12–15, 2006. (Michael Lagunoff lab)

73. Lagos D, Trotter MW, Vart RJ, Wang HW, Matthews NC, Hansen A, Gotch F, Boshoff C. Kaposi Sarcoma Herpesvirus Employs Transcriptional Mechanisms to Regulate Antigen Presentation in Lymphatic Endothelial Cells. Accepted for oral presentation (abstract 64) at 9th Annual KSHV Workshop, Hyannis, MA. July 12–15, 2006.

74. Lamers SL, Galligan D, Zhao L, Yu S, Shagrun L, De Oliveria T, Salemi M, McGrath MS. Multi-site tissue autopsy samples from patients with dementia and AIDS related lymphoma show distinct patterns of HIV evolution in vivo. Poster presentation at the XVI International AIDS Conference in Toronto ON. August 13–18, 2006

75. Nohle DG, Hackman BA, Ayers LW. Software-assisted visual analysis and review of tissue microarray (TMA) images facilitated by XML. Poster presentation at APIII 2006 11th Annual Conference, Vancouver BC, August 15–18, 2006.

76. Ayers LW, Hackman BA, McGrath MS, Silver S, Orenstein J, Bhatia K. HIV/AIDS-related Lymphoma Immunophenotypes in Biopsy Samples from Patients During Pre and Post HAART Treatment Periods. Presented to 10th International Conference on Malignancies in AIDS and Other Acquired Immunodeficiencies: Basic, Epidemiologic and Clinical Research, North Bethesda, MD, October 16–17 2006.

77. Shagrun LS, Morris A, McGrath MS. "ProMacs" in cases of HIV+ and HIV- Large Cell Lymphoma. Presented to 10th International Conference on Malignancies in AIDS and Other Acquired Immunodeficiencies: Basic, Epidemiologic and Clinical Research, North Bethesda, MD, October 16–17 2006.

78. Mwanda OW, Orem J, Fu P, Banura C, Kakembo J, Ness A, Johnson J, Subbiah V, Bako J, Black J, Katongole-Mbidde E, Remick SC. Dose modified oral chemotherapy for AIDS-related non-Hodgkin's Lymphoma (AR-NHL) in East Africa: Impact on CD4+ count and HIV-1 replication; and retrospective look at similar regimen in pre-HAART era in the USA. AB# 13 presented to 10^th ^International Conference on Malignancies in AIDS and Other Acquired Immunodeficiencies in North Bethesda, Maryland; October 16–17, 2006.

79. Mwanda OW, Orem J, Fu P, Banura C, Kakembo J, Ness A, Johnson J, Subbiah V, Bako J, Black J, Katongole-Mbidde E, Remick SC. Dose modified oral chemotherapy for AIDS-related non-Hodgkin's Lymphoma (AR-NHL) in East Africa. AB# 47 presented to 10^th ^International Conference on Malignancies in AIDS and Other Acquired Immunodeficiencies in North Bethesda, Maryland; October 16–17, 2006.

80. Bako J, Mwanda OW, Orem J, Fu P, Subbiah V, Banura C, Kakembo J, Ness A, Johnson J, Katongole-Mbidde E, Remick SC. Comparative retrospective study of patients at diagnosis with AIDS-related non-Hodgkin's Lymphoma (AR-NHL) treated with oral chemotherapy in the pre-HAART era in East Africa and the USA. AB# 48 presented to 10^th ^International Conference on Malignancies in AIDS and Other Acquired Immunodeficiencies in North Bethesda, Maryland; October 16–17, 2006.

81. Subbiah V, Mwanda OW, Orem J, Fu P, Banura C, Kakembo J, Ness A, Johnson J, Bako J, Katongole-Mbidde E, Remick SC. Analyses of prognostic factors for survival in East African patients with AIDS-related non-Hodgkin's Lymphoma (AR-NHL) treated with dose modified oral chemotherapy. AB# 50 presented to 10^th ^International Conference on Malignancies in AIDS and Other Acquired Immunodeficiencies in North Bethesda, Maryland; October 16–17, 2006.

82. Orem J, Mwanda OW, Fu P, Banura C, Kakembo J, Ness A, Johnson J, Subbiah V, Bako J, Katongole-Mbidde E, Remick SC. Implications of oral chemotherapy for AIDS-related non-Hodgkin's lymphoma (AR-NHL). AB# 51 presented to 10^th ^International Conference on Malignancies in AIDS and Other Acquired Immunodeficiencies in North Bethesda, Maryland; October 16–17, 2006.

83. Zhang RZ, Miller RG, Gascon R, Gelinas DF, Katz J, Mass J, Scholtz D, Lancero M, Narvaez A, McGrath MS. Evidence for abnormal monocyte immunoglobulin receptor expression in sporadic amyotrophic lateral sclerosis (sALS). Accepted for Poster presentation at the 17th International Symposium on ALS/MND in Yokohama, Japan, November 30 – December 2, 2006

## Notes

***Grant Support: ***National Cancer Institute (NCI), U01-CA66529, U01-CA96230, and U01-CA066531

***Requests for reprints: ***Debra Garcia, ACSR CODCC, 995 Potrero Avenue, Building 80, Ward 84, San Francisco, CA 94110
